# Combined VEGFR and MAPK pathway inhibition in angiosarcoma

**DOI:** 10.1038/s41598-021-88703-9

**Published:** 2021-04-30

**Authors:** Michael J. Wagner, Yasmin A. Lyons, Jean H. Siedel, Robert Dood, Archana S. Nagaraja, Monika Haemmerle, Lingegowda S. Mangala, Pritha Chanana, Alexander J. Lazar, Wei-Lien Wang, Vinod Ravi, Eric C. Holland, Anil K. Sood

**Affiliations:** 1 Division of Medical Oncology, University of Washington, 825 Eastlake Ave E, Seattle, WA 98109 USA; 2 Clinical Research Division, Fred Hutchinson Cancer Research Center, Seattle, USA; 3 Department of Gynecologic Oncology and Reproductive Medicine and Center for RNA Interference and Non-Coding RNA, UT MD Anderson Cancer Center, Houston, USA; 4 Bioinformatics Shared Resource, Fred Hutchinson Cancer Research Center, Seattle, USA; 5 Department of Pathology, UT MD Anderson Cancer Center, Houston, USA; 6 Sarcoma Medical Oncology, UT MD Anderson Cancer Center, Houston, USA; 7 Division of Human Biology, Fred Hutchinson Cancer Research Center, Seattle, USA; 8 Present Address: Section for Experimental Pathology, Medical Faculty, Institute of Pathology, Martin-Luther University Halle-Wittenberg, Halle (Saale), Germany; 9 Department of Genomic Medicine, UT MD Anderson Cancer Center, Houston, USA

**Keywords:** Cancer therapy, Sarcoma

## Abstract

Angiosarcoma is an aggressive malignancy of endothelial cells that carries a high mortality rate. Cytotoxic chemotherapy can elicit clinical responses, but the duration of response is limited. Sequencing reveals multiple mutations in angiogenesis pathways in angiosarcomas, particularly in vascular endothelial growth factor (VEGFR) and mitogen-activated protein kinase (MAPK) signaling. We aimed to determine the biological relevance of these pathways in angiosarcoma. Tissue microarray consisting of clinical formalin-fixed paraffin embedded tissue archival samples were stained for phospho- extracellular signal-regulated kinase (p-ERK) with immunohistochemistry. Angiosarcoma cell lines were treated with the mitogen-activated protein kinase kinase (MEK) inhibitor trametinib, pan-VEGFR inhibitor cediranib, or combined trametinib and cediranib and viability was assessed. Reverse phase protein array (RPPA) was performed to assess multiple oncogenic protein pathways. SVR angiosarcoma cells were grown in vivo and gene expression effects of treatment were assessed with whole exome RNA sequencing. MAPK signaling was found active in over half of clinical angiosarcoma samples. Inhibition of MAPK signaling with the MEK inhibitor trametinib decreased the viability of angiosarcoma cells. Combined inhibition of the VEGF and MAPK pathways with cediranib and trametinib had an additive effect in in vitro models, and a combinatorial effect in an in vivo model. Combined treatment led to smaller tumors than treatment with either agent alone. RNA-seq demonstrated distinct expression signatures between the trametinib treated tumors and those treated with both trametinib and cediranib. These results indicate a clinical study of combined VEGFR and MEK inhibition in angiosarcoma is warranted.

## Introduction

Angiosarcoma is a rare malignancy of endothelial cells, with an incidence of 400–500 new cases per year in the United States. They are aggressive tumors with a high propensity to spread. Five year survival for all patients, including those presenting with localized disease is less than 50%, and median overall survival for patients with metastatic disease is eight months^1^. Therefore, new treatment approaches are needed.

Initial efforts to identify drivers of angiosarcoma have revealed several recurrent aberrations in angiogenesis pathways^2,3^. These include activating mutations in kinase insert domain receptor (*KDR)* and phospholipase C gamma 1 (*PLCG1)*, and loss of function mutation in receptor-type tyrosine-protein phosphatase beta (*PTPRB)*^4,5^. Secondary angiosarcomas (e.g., radiation and lymphedema induced) are characterized by Myc proto-oncogene *(MYC)* amplification and a subset also have FMS related receptor tyrosine kinase 4 (*FLT4)* amplification^6^. This greater understanding of driver mutations in angiosarcoma yields insights into potential targets for clinical care. Some analyses have suggested alterations in pathways downstream from vascular endothelial growth factor receptor 2 (VEGFR2) are relevant targets for angiosarcoma, focusing largely on the Phosphoinositide-3-Kinase (PI3K)/ mechanistic target of rapamycin (mTOR) and mitogen-activated protein kinase (MAPK) pathways. In particular, MAPK pathway alterations have been identified in angiosarcoma clinical samples with mutations in K-Ras Proto-Oncogene (*KRAS)*, H-Ras Proto-Oncogene (*HRAS)*, N-Ras Proto-Oncogene (*NRAS)*, B-Raf Proto-Oncogene (*BRAF)*, Raf-1 Proto-Oncogene (*CRAF)*, mitogen-activated protein kinase 1 (*MAPK1)* and Neurofibromin 1 (*NF1)*^7^*.* The recurrent R707Q mutation in PLCG1 leads to canonical activation of the MAPK pathway^8^ and leads to acquired resistance to VEGFR inhibition^9^.

In spite of these potentially targetable mutations, targeted agents modulating angiogenesis pathways in angiosarcoma clinical studies have overall been disappointing with response rates less than 20%^10–12^. One potential strategy to overcome this intrinsic resistance is to concurrently target multiple parallel signaling pathways. Indeed, dual inhibition of mTOR and MEK has been shown to be efficacious in a mouse model of angiosarcoma^13^. We sought to further investigate the potential for targeting the MAPK pathway in angiosarcoma with dual inhibition of the VEGFR-MAPK axis.

## Materials and methods

### Cell lines and cell culture

SVR cells were obtained from ATCC and were maintained in Dulbecco's Modified Eagle Medium (DMEM) + 5% FBS + 0.1% gentamicin sulfate (Gemini Bioproducts, Calabasas, CA). ASM-1 angiosarcoma cells were generously provided by Dr. James Kirkpatrick (Johannes Gutenberg-Universität Mainz, Germany)^14^. HAMON angiosarcoma cells were generously provided by Dr. Riichiro Abe (Hokkaido University, Japan)^15^. ASM-1 and HAMON cells were maintained in EGM-2 endothelial cell growth media (Lonza). All cells were screened for mycoplasma and experiments were performed at 60–80% confluence.

### Drugs

Cediranib, a VEGFR inhibitor, and trametinib, a MEK inhibitor, were obtained from Selleckchem. They were reconstituted in DMSO and aliquots were stored at -20 degrees Celsius until use.

### Immunohistochemistry

Unstained slide was prepared from a tissue microarray consisting of sixty-nine clinical formalin-fixed paraffin-embedded archival angiosarcoma specimens. IHC was performed on a Leica BOND RX automated IHC stainer (Lecia Biosystems, Buffalo Groove, IL). The slide was baked at 60 °C, dewaxed with Bond dewax solution at 72 °C, rinsed in 100% EtOH, and Bond washed. Citrate buffer was used for epitope retrieval at 100 °C. Slides were blocked with hydrogen peroxide (3.0% H_2_O_2_) for ten minutes and then with protein block. Primary antibody against phospho-ERK (p44/42 MAPK 1:150 dilution, Cell Signaling Cat # 4370), the active form of ERK, was applied for 15 min at room temperature. Polymer enhanced secondary antibody Poly-HRP anti-rabbit IgG was applied for eight minutes. Labeling was assessed for degree of nuclear intensity (weak, moderate or strong) and extent of labeling (0, 0%; 1 + , 5%; 2 + , 6–25%; 3 + , 26–50%; 4 + , 51–75%; 5 + , > 75%). Specimens were labeled as being positive phospho-ERK when having at least 2 + tumoral labeling extent of any intensity. Focal labeling was defined as 1 + extent of any intensity. Clinical annotation for survival status was performed.

### Viability assays

SVR, HAMON, or ASM-1 cells were seeded in a 96 well plate at 5000 cells per well. Cells were grown overnight to 60–80% confluence and drugs were added the next day. Viability was assessed via standard MTT assay as previously described^16^. Viability was assessed at 96 h for SVR cells and 7 days for HAMON and ASM-1 cells (2 doubling times). Media and drug were changed after 4 days of treatment for the prolonged treatment.

### Animal studies

8–12 week old athymic nude mice were obtained from Taconic Farms (Hudson, NY). All experimental protocols were approved by The University of Texas MD Anderson Cancer Center Institutional Animal Care and Use Committee (IACUC) or the Fred Hutchinson Cancer Center IACUC as applicable for the location where the experiment was performed. All experiments done on animals were in accordance with guidelines of the American Association for Accreditation of Laboratory Animal Care and the US Public Health Service Policy on Human Care and Use of Laboratory Animals. 20,000 SVR cells were injected subcutaneously into the flank of each mouse on day 0. Tumors were allowed to establish for 3–7 days and then treatment began by oral gavage. Mice were treated with vehicle (10% Cremaphor, 10% PEG 400, 1% tween), trametinib 1 mg/kg, cediranib 3 mg/kg, or trametinib and cediranib combined by oral gavage daily until tumors became ulcerated or greater than 1 cm. Tumor volume was calculated as (4/3)π × (w/2)^2^ (l/2) where w is tumor width and l is tumor length as assessed with calipers. For tumor weight measurements, tumors were harvested after 17 days of treatment. Mice were euthanized with cervical dislocation and CO_2_ inhalation via compressed gas as per IACUC approved protocols. Data are presented as mean ± standard error.

### Statistical design

Differences in continuous variables were compared using Student’s t test or analysis of variance (ANOVA) using GraphPad Prism 7. Survival was assessed using the Kaplan Meier method and log rank test in SPSS (statistical software, IBM SPSS Statistics Version 22, Armonk, NY, https://www.ibm.com/). A p-value of < 0.05 was considered statistically significant.

### Reverse phase protein array

HAMON cells were cultured in 60 mm dishes and treated for 72 h with vehicle, trametinib 100 nm, cediranib 4 µM, or combined trametinib 100 nM and cediranib 4 µM. Protein lysates were generated as per previously published protocols and submitted to the RPPA core facility at MD Anderson Cancer Center for analysis as previously described^17^.

### RNA isolation

Total RNA extraction from flash frozen mouse tumor tissue was done using the RNEasy Mini kit (Qiagen, Valencia, CA). The method involved using 5 mm stainless beads in conjunction with the TissueLyser LT (Qiagen, Valencia, CA) to achieve tissue lysis and homogenization. On-column DNase I digestion and RNA extraction was carried out following the manufacturer’s recommended protocol. Total RNA integrity was checked using an Agilent 4200 TapeStation (Agilent Technologies, Inc., Santa Clara, CA) and quantified using a Trinean DropSense96 spectrophotometer (Caliper Life Sciences, Hopkinton, MA).

### RNA-seq expression analysis

RNA was extracted from frozen xenograft tumor samples after treatment with vehicle, trametinib, cediranib, or combined trametinib and cediranib as described above and whole exome RNA sequencing was performed. This method of RNA sequencing was selected due to relatively high rates of RNA degradation in the tumor samples^18^. TruSeq RNA Exome library prep kits (Illumina, Inc., San Diego, CA, USA) were used to prepare RNA-seq libraries. Agilent 4200 TapeStation (Agilent Technologies, Santa Clara, CA, USA) was used to validate library size distribution. Life Technologies’ Invitrogen Qubit 2.0 Fluorometer (Life Technologies-Invitrogen, Carlsbad, CA, USA) was used for library QC, to blend pooled indexed libraries, and for cluster optimization. Libraries were pooled 12-plex and were clustered onto one flow cell lane. For sequencing, an Illumina HiSeq 2500 in rapid mode with a paired-end, 50 base read length (PE50) was used. Illumina's Real Time Analysis v1.18 software was used for image analysis and base calling. This was followed by demultiplexing of indexed reads and generation of FASTQ files, using bcl2fastq Conversion Software v1.8.4 from Illumina (http://support.illumina.com/downloads/bcl2fastq_conversion_software_184.html).

Alignment was performed against the GRCm38 reference genome using STAR v-2.7.1a in the two-pass alignment mode^19^. Alignment quality control metrics like gene body coverage, inner distance and read duplication were generated using the RSeQC package v-3.0.0^20^. Subread featureCounts v-1.6.0 was used to perform gene expression quantification and generate counts^21^, followed by Bioconductor edgeR to perform the differential expression analysis between various groups^22,23^. A significance threshold of log2FC ≥ 1.5 or log2FC ≤ − 1.5 at 5% FDR was used to define the genes of interest in each comparison.

Gene set enrichment analysis (GSEA) was performed as previously described and used all 9 MSigDb collections (C1 through C8 and H) for the analysis and the annotations from the software provided^24^. Mouse_ENSEMBL_Gene_ID_Human_Orthologs_MSigDB.v7.2.chip file were used for the transformation of mouse data to human symbols. Additionally the publicly available tool Webgestalt^25^ was also used to perform pathway enrichment analysis against multiple databases such as KEGG^26^, Reactome^27^, and Panther^28^.

## Results

### p-ERK expression in human angiosarcoma samples

**Figure 1:**
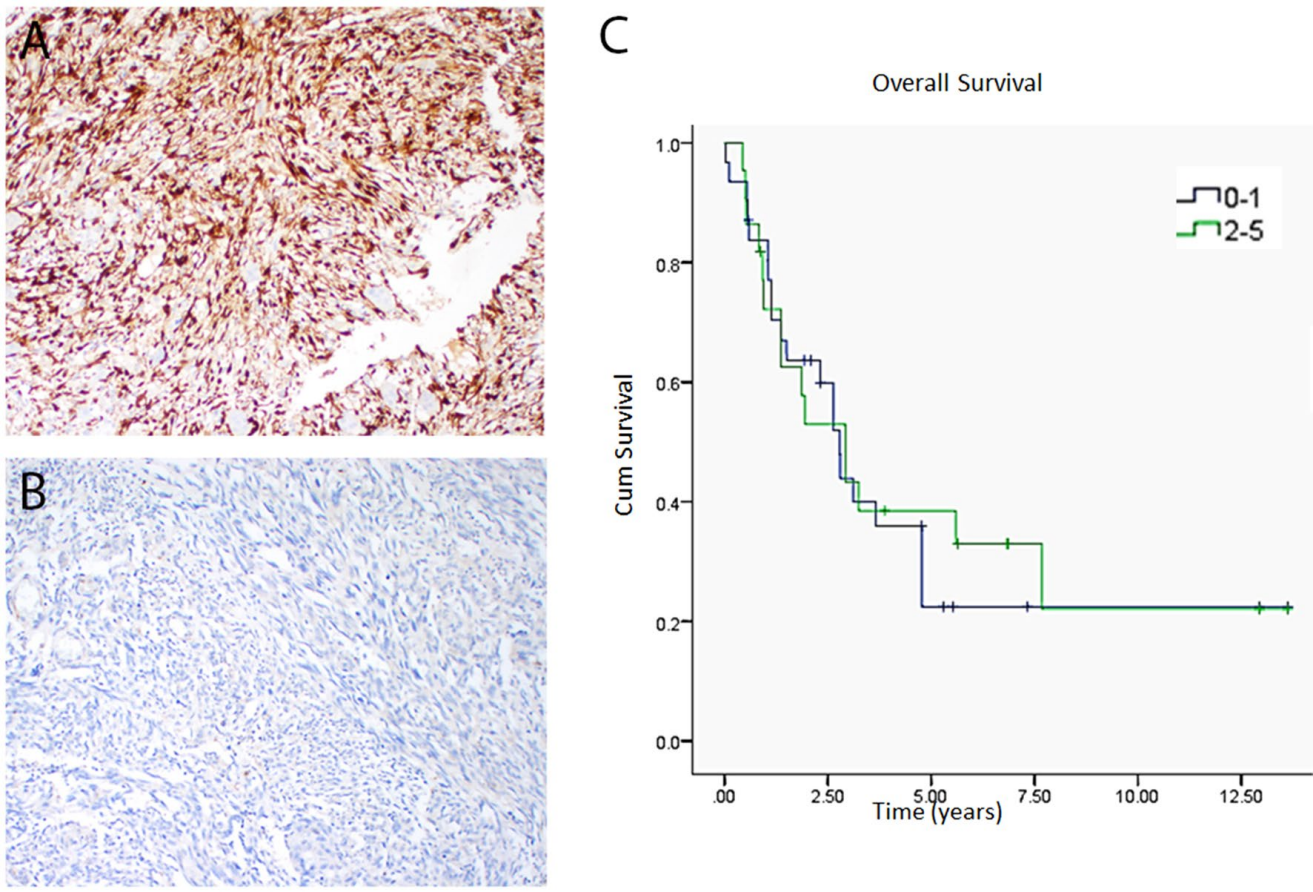
p-ERK staining in clinical angiosarcoma samples and impact on survival. A tissue microarray with 60 unique clinically annotated angiosarcoma patient samples was stained for phospho-ERK. 26 (44%) stained positive, 27 (45%) stained negative, and 7 (12%) had focal staining (**A**,**B**). There was no correlation between phospho-ERK staining and overall survival between cases with low (0–1) phospho-ERK and high (2–5) phospho-ERK (**C**, p = 0.88). Unedited images were formatted in Adobe Photoshop 2020 (https://www.adobe.com/) to create the figure layout. Survival was assessed using the Kaplan Meier method and log rank test in SPSS (statistical software, IBM SPSS Statistics Version 22, Armonk, NY, https://www.ibm.com/).

We first sought to investigate if the MAPK pathway is activated in clinical angiosarcoma samples. Sixty out of sixty-nine cores were analyzable. Twenty six (44%) samples were considered to be positive for p-ERK (> 5%) and typically exhibited moderate to strong nuclear labeling intensity. Concurrent cytoplasmic labeling was also seen. Seven cases (12%) had focal labeling (≤ 5%) and 27 (45%) were negative (Fig. 1A,B). Survival analysis reveals no statistically significant correlation between phospho-ERK staining and overall survival (Fig. 1C, p = 0.88).

### MEK inhibition and VEGFR inhibition were not synergistic in vitro

**Figure 2:**
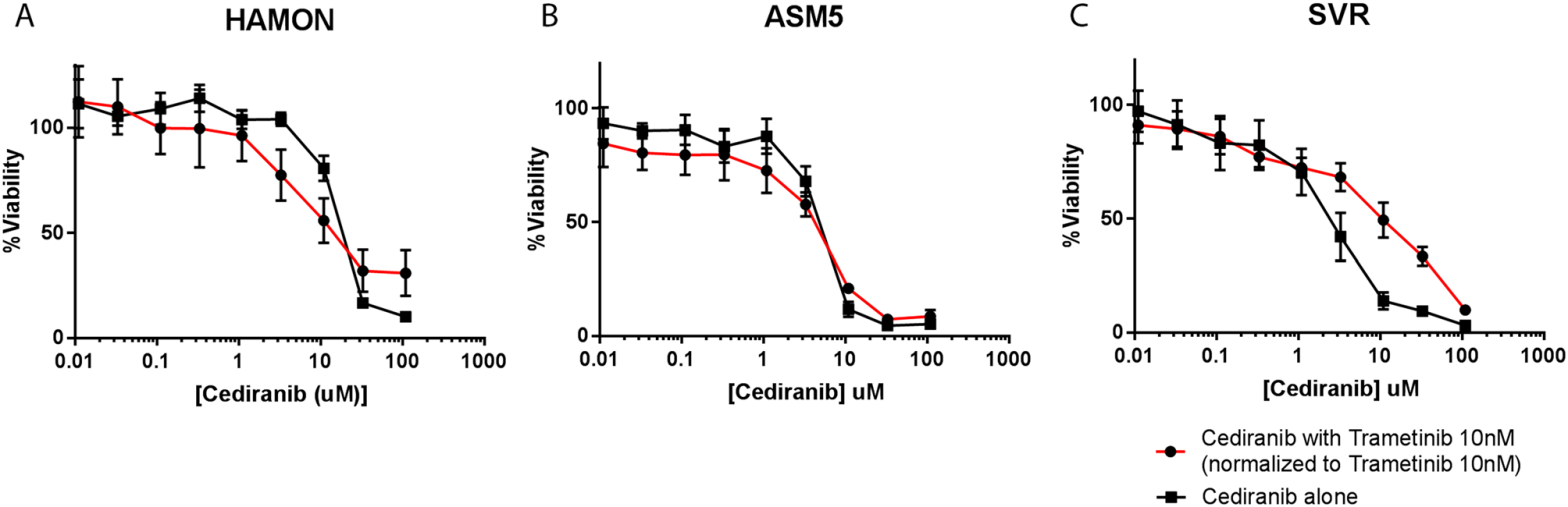
Lack of synergy between trametinib and cediranib in angiosarcoma cell lines in vitro. HAMON (**A**), ASM5 (**B**), and SVR (**C**) angiosarcoma cells were grown in increasing concentrations of cediranib, either alone or in combination with 10 nM trametinib. With 10 nM trametinib alone, HAMON cells had 37% viability, ASM5 had 63% viability, and SVR had 44% viability in the experiments presented. When normalized for the single agent activity of 10 nM trametinib, there was no synergy between cediranib and trametinib in any of the models. Graphs were made in GraphPad Prism 7 (https://www.graphpad.com/scientific-software/prism/). Unedited/cropped images were formatted in Adobe Photoshop 2020 (https://www.adobe.com/) to create the figure layout.

To test the *in* vitro sensitivity of angiosarcoma cell lines to MAPK inhibition (trametinib), VEGFR inhibition (cediranib), and combined MAPK and VEGFR inhibition (combination of trametinib and cediranib), we used the MTT assay. Although there was single agent activity with either drug, the addition of trametinib did not increase the effects of cediranib in vitro (Fig. 2). For HAMON, IC50 with cediranib alone was 20.2 µM (95% CI 15.2–27.0) and 16.7 µM (95% CI 10.9–25.7) with the addition of 10 µM trametinib. For ASM5 cells, IC50 with cediranib alone was 4 µM (95%CI 3.3–5.2) and 3.16 µM (95%CI: 3.3–4.4) with the addition of 10 nM trametinib to cediranib. For SVR cells, IC50 with cediranib alone was 2.2 µM (95% CI 1.8–2.7) and 8.8 µM (95% CI 6.2–12.6) with the addition of 10 nM trametinib to cediranib.

### Combined VEGFR and MEK inhibition delays SVR tumor growth in an in vivo model

**Figure 3:**
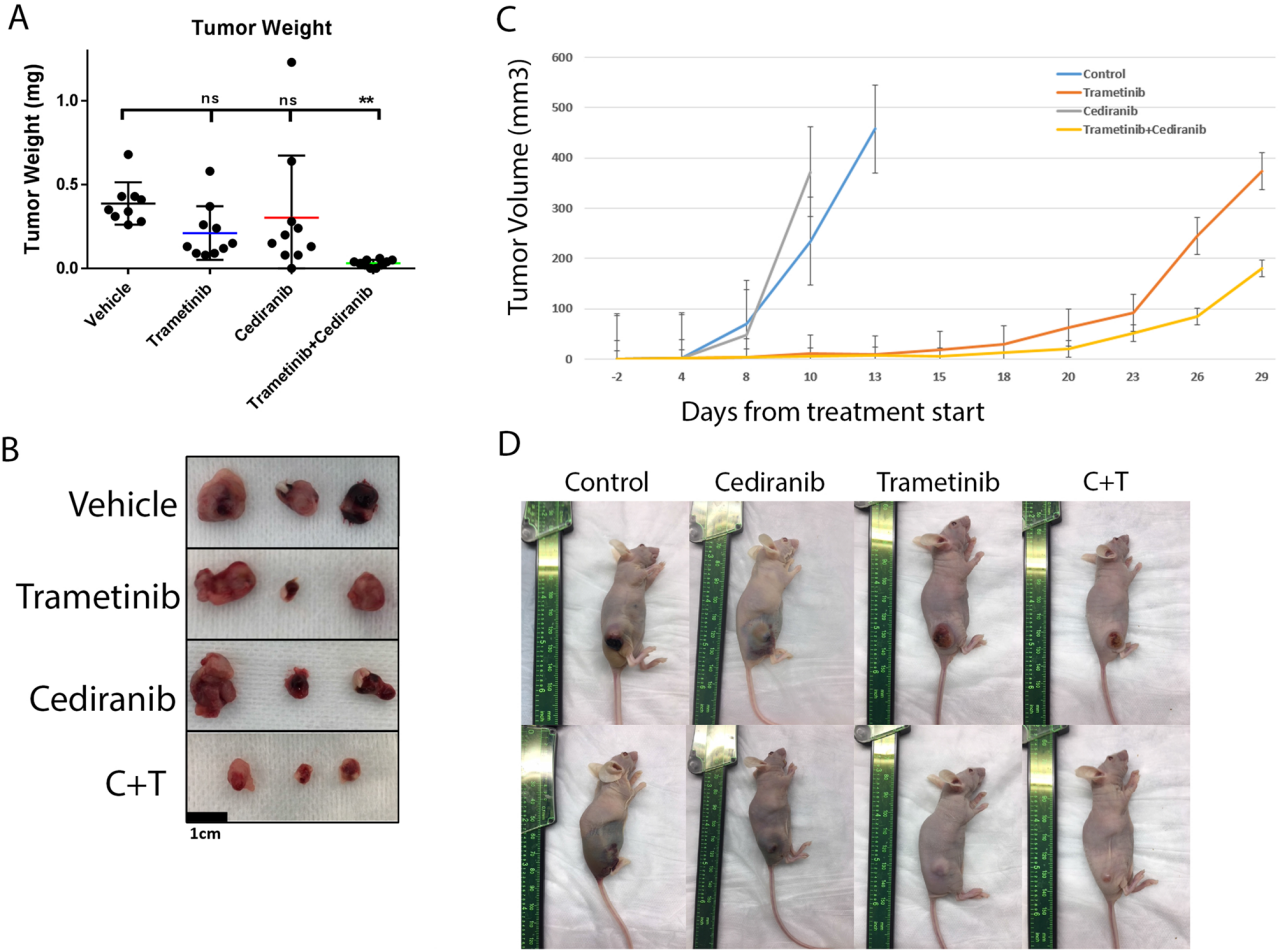
Combinatorial effect of trametinib and cediranib on SVR xenografts. Two treatment experiments were performed with different endpoints. In one experiment (left, **A**,**B**) all tumors were harvested at the same timepoint to allow for comparison of tumor weight between groups. In the second (right, **C**,**D**), tumors were harvested after they grew above the IACUC mandated maximum size or became ulcerated. (**A**) Combined treatment with trametinib and cediranib resulted in decreased tumor weight. (**B**) Representative tumors after 17 days of treatment. (**C**) Tumor growth curves of SVR xenografts treated with vehicle, trametinib, cediranib, or cediranib combined with trametinib in a separate experiment in which trametinib and combination treated mice were allowed to grow after control and cediranib treated mice were sacrificed. Curves discontinue when any mice from the representative group had to be sacrificed. (**D**) Representative mice from each group (two representative pictures from each group) demonstrating tumor hemorrhage in the control and cediranib groups and not in the trametinib treated groups. Data are presented ± SEM. Graphs were made in GraphPad Prism 7 (https://www.graphpad.com/scientific-software/prism/). Unedited/cropped images were formatted in Adobe Photoshop 2020 (https://www.adobe.com/) to create the figure layout.

We then assessed the effects of trametinib and cediranib in an in vivo model. SVR cells were chosen due to their ability to form tumors when injected subcutaneously in nude mice. Since a significant percentage of angiosarcomas arise in the skin, this is considered an orthotopic site. SVR cells were injected subcutaneously into the flanks of nude mice. Tumors were allowed to establish and then the mice were treated daily with cediranib, trametinib, cediranib and trametinib combined, or vehicle. Control and cediranib treated mice had rapid tumor growth (Fig. 3C) and developed hemorrhagic tumors as evidenced by peritumoral ecchymosis (Fig. 3D). Trametinib treated mice took longer for tumors to grow and did not develop hemorrhage. By day 23 after treatment initiation, all of the control and cediranib treated mice were sacrificed due to tumor growth or ulceration. Combination treated mice had tumors that were significantly smaller than those treated with single agent trametinib by day 26 after treatment (p = 0.04).

A separate experiment was performed under the same conditions in order to evaluate tumors at the same time point on treatment. All mice were sacrificed after 17 days of treatment. In vivo tumor size was reduced by > 90% in the combination group compared to vehicle or either drug alone at (vehicle 478 ± 136 mm^3^, trametinib 127 ± 51.4 mm^3^, cediranib 244 ± 109 mm^3^, and trametinib + cediranib 7.6 ± 2.2 mm^3^; ANOVA p < 0.01). There was also a similar reduction in tumor weight (vehicle 0.39 ± 0.04 g, trametinib 0.21 ± 0.05 g, cediranib 0.30 ± 0.12 g, and trametinib + cediranib 0.03 ± 0.01 g; p < 0.01) (Fig. 3A,B), and a significant decrease in proliferation in the combination group compared with either treatment alone as assessed by Ki67 immunohistochemistry (Supp Fig. S1A,B). There were no statistically significant changes in cleaved caspase 3 in any of the in vivo treated tumors, suggesting that the observed effect is not by induction of apoptosis in the in vivo model (Supp Fig. S1A,B).

### RPPA analysis of HAMON cells demonstrates parallel pathway effects of VEGFR and MAPK inhibition

**Figure 4:**
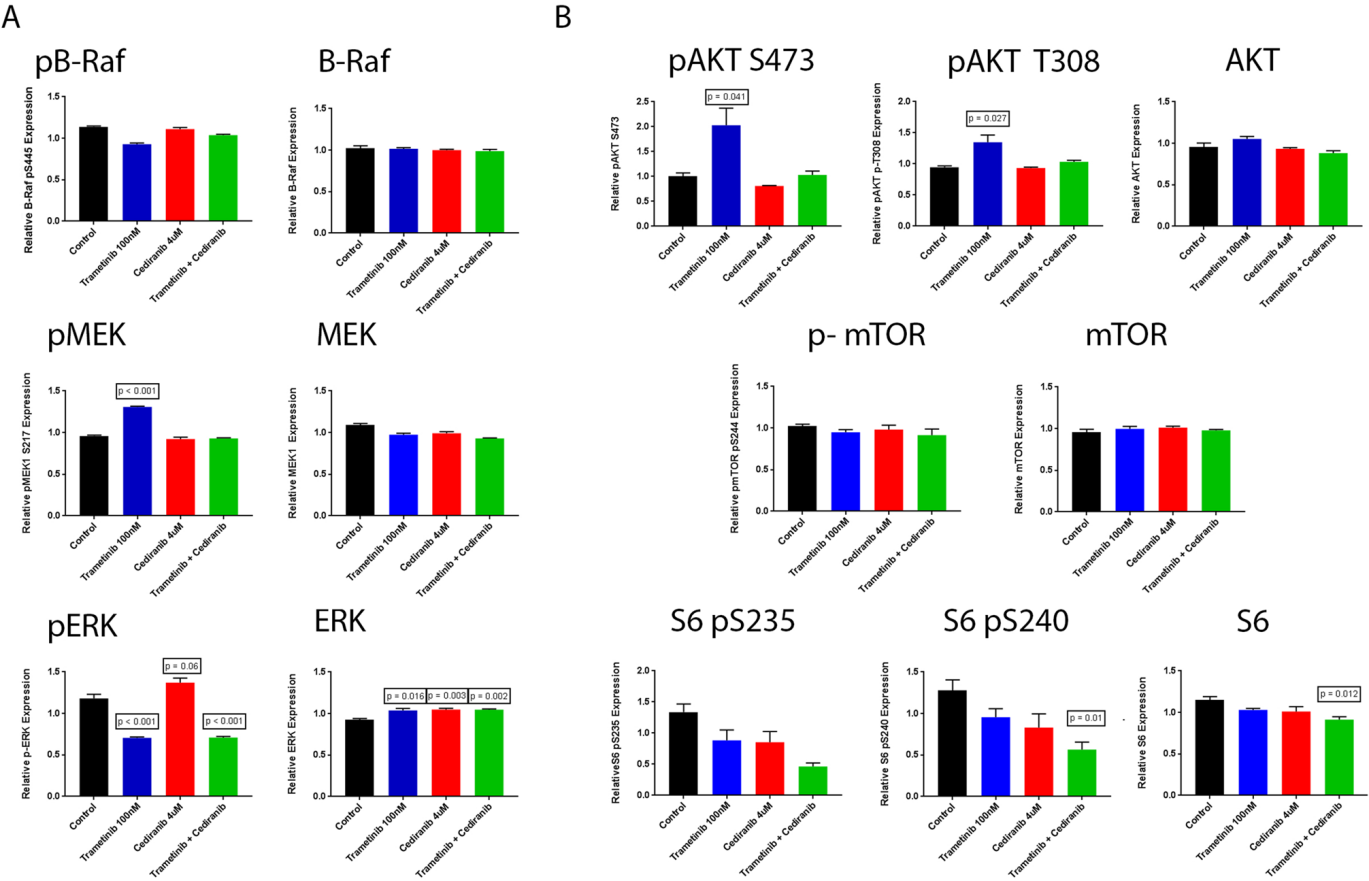
Reverse phase protein array (RPPA) analysis of HAMON cells treated with Trametinib, Cediranib, and the combination of Trametinib and Cediranib for 72 h. (**A**) Relative expression of MAPK related proteins and phospho-proteins. (**B**) Relative expression of AKT/mTOR pathway proteins. Statistically significant comparisons to control are noted, defined as t-test p < 0.05. Other comparisons of treatment to control were not statistically significant. Graphs were made in GraphPad Prism 7 (https://www.graphpad.com/scientific-software/prism/). Unedited/cropped images were formatted in Adobe Photoshop 2020 (https://www.adobe.com/) to create the figure layout.

HAMON angiosarcoma cells, chosen because they are a patient derived cell line, were treated for 72 h and protein lysates were analyzed by RPPA. Although it did not reach statistical significance, phosphorylated ERK trended towards an increase in cediranib treated cells, and this was suppressed with the addition of trametinib (Fig. 4A). Additionally, a paradoxical increase in MEK phosphorylation with trametinib treatment alone was observed, which was inhibited with the addition of cediranib (Fig. 4A). No changes were observed in BRAF phosphorylation or expression (Fig. 4A). Similarly, Protein kinase B (AKT) phosphorylation increased with MEKi alone, and this increase was suppressed by cediranib. There appeared to be an additive decrease in S6 phosphorylation with trametinib and cediranib treatment (Fig. 4B). Apoptosis associated proteins were increased in combination treatment and mitosis related proteins were decreased in combination treatment (Supplemental Fig. S2). Western blot analysis of p-ERK and p-AKT in three angiosarcoma cell lines suggested slight increases in p-ERK with cediranib treatment and slight increases in p-AKT with effective trametinib treatment (supplemental Fig. S3), similar to what was seen in the RPPA analysis.

### Transcriptional analysis reveals distinct gene expression profiles with MAPK and VEGFR inhibition

**Figure 5:**
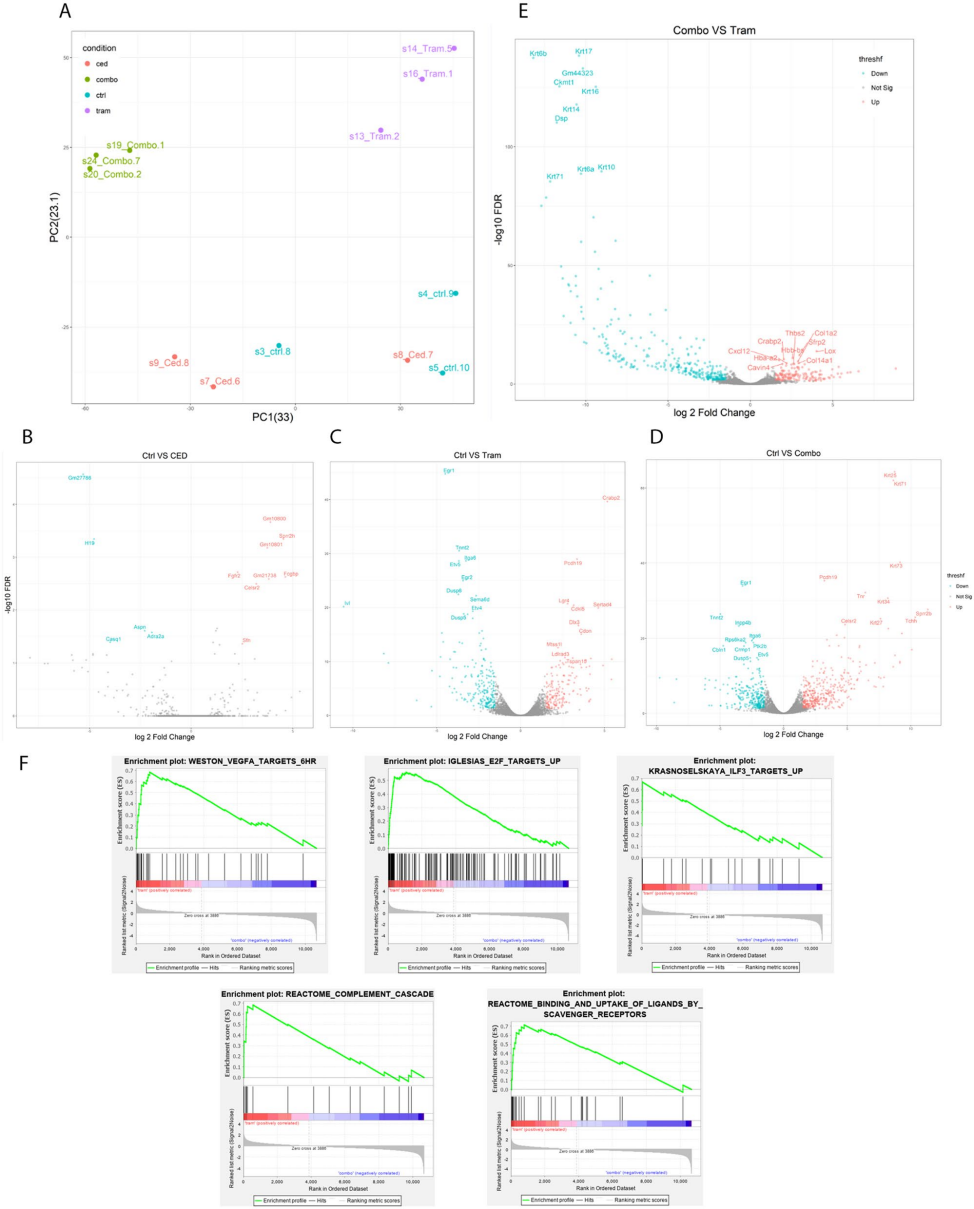
RNA sequencing of SVR xenograft whole lysate after treatment. (**A**) Principal component analysis demonstrates 3 distinct patterns of expression, with control (blue) and cediranib (red) treated tumors in one cluster, and trametinib treated tumors (purple) and combined trametinib and cediranib treated tumors (green) clustering separately. (**B**–**D**) Volcano plots for cediranib (**B**), trametinib (**C**), and combination (**D**) treated tumors compared to controls vehicle treated tumors. (**E**) Volcano plot comparing trametinib treated tumors to combination treated tumors. (**F**) Gene sets enriched in trametinib treated tumors compared with combination treated tumors. Volcano plots were made in R (https://www.r-project.org/). Graphs in panel (**F**) were made with GSEA software available at https://www.gsea-msigdb.org/gsea/index.jsp.

Expression profiles for cediranib treated tumors were similar to that of untreated control. Expression profiles of trametinib treated tumor and combined trametinib and cediranib treated tumors were distinct in principal component analysis (PCA), and separate from control or cediranib treated tumors (Fig. 5A). When compared to control, genes significantly upregulated in the tumors treated with cediranib alone included fibroblast growth factor receptor 2 (FGFR2) and Fc fragment of IgG binding protein (FCGBP) (Fig. 5B). Trametinib treatment led to lower expression levels of members of the dual specificity protein phosphatase (DUSP) subfamily DUSP5 and DUSP6, integrin subunit alpha 6 (ITGA6), ETS variant transcription factor 5 (ETV5), and early growth response 1 (EGR1), and higher levels of protocadherin 19 (PCDH19) compared to untreated tumors (Fig. 5C,D). Both trametinib and combined treated tumors had high levels of transcripts associated with skin such as Leucine-rich repeat-containing G-protein coupled receptor 4 (LGR4), SERTA domain-containing protein 4 (SERTAD4), and keratins (KRT) (Fig. 5C,D), consistent with the infiltrative growth pattern seen in these cutaneous tumors. Comparison of combination treated tumors with trametinib treated tumors demonstrated higher keratin levels in the combination treated tumors (Fig. 5E).

Gene set enrichment analysis (GSEA) revealed that, compared to trametinib treated tumors, control tumors were enriched for MAPK pathway related gene sets, phagosome acidification pathway, Interleukin 2 (IL2), Interleukin 4 (IL4), Interleukin 7 (IL7), and phospholipid metabolic processes. In the comparison of trametinib treated tumors vs combination treated tumors, trametinib treated tumors were most enriched for E2F transcription factor (E2F) targets (previously associated with angiosarcoma tumor^29^), VEGF-A signaling, and innate immune pathways including upregulation of targets of interleukin enhancer binding factor 3 (ILF3), complement cascade activation and increased activity of scavenger receptors with a nominal p- value < 0.001 (Fig. 5F).

In the GSEA by WebGestalt, angiogenesis and WNT signaling pathway related genes were upregulated in trametinib treated tumors compared to control and control tumors were enriched for MAPK pathway activation. When compared to combination treated tumors, trametinib treated tumors were enriched for gene sets related to muscle, extracellular matrix organization, and WNT signaling activation. Compared to trametinib treated tumors, combination treated tumors were enriched for apoptosis related gene sets and keratinization.

## Discussion

Despite recent advances in cancer therapy, outcomes for patients with angiosarcoma remain poor. Recent data has suggested a role for dysregulated angiogenic signaling as a driver for angiosarcoma, but angiogenesis inhibitors overall had disappointing results in the clinic. Meaningful responses seem to be limited to patients with alterations in the VEGF receptors themselves^4,30^. Multi-pathway inhibition has proven to be a successful strategy in multiple cancer types in overcoming intrinsic and acquired resistance to inhibition of a single oncogenic signaling pathway^31,32^.

In recent years, sequencing efforts in angiosarcoma revealed aberrations in MAPK signaling. We hypothesized that MAPK signaling would be active in clinical angiosarcoma samples, and that targeting the MAPK pathway would be effective against angiosarcoma cells. Indeed, we found that MAPK activation was present by IHC in over half of clinical angiosarcoma samples and that treatment with either the MEK inhibitor trametinib or pan-VEGFR inhibitor cediranib decreased the viability of cultured angiosarcoma cell lines. Interestingly, although there was no synergy with the combined treatments in vitro, there was a combinatorial effect noted in the in vivo model. The unexpected discrepancy between the in vitro assays and in vivo results suggests that the microenvironment plays an important role in maintaining angiosarcoma tumors.

To date, little is known about the microenvironment of angiosarcoma. Since angiosarcoma tumor cells also stain positive for endothelial cell markers, clear delineation of the normal endothelium and blood supply cannot be assessed using typical endothelial cell markers by IHC due to their shared lineage. The few cases of remarkable responders to angiogenesis inhibitors where translational correlative data attribute the responses to alterations in angiogenesis genes such as KDR in the tumor cells rather than affecting the normal endothelial cells in the microenvironement^4,30^. One hypothesis to explain the different results in this study between in vitro and in vivo models is that normal endothelial cells in the microenvironment are also being targeted, as both MAPK and mTOR signaling are important in physiologic angiogenesis. However, efforts to target physiologic angiogenesis in angiosarcoma clinically have been largely unsuccessful^10–12^.

We performed gene set enrichment analysis to assess key pathways that may be differentially expressed as a result of treatment. As expected, the tumors that were not treated with trametinib had enrichment in MAPK pathway related gene sets. WNT signaling was enriched in trametinib treated tumors, consistent with the finding that MEK inhibition increases WNT signaling in colon cancer cells serving as a possible escape mechanism for colon cancer cells in the face of MEK inhibition^33^. The increase in angiogenesis pathways in trametinib treated cells in the GSEA is consistent with the mild increase in mTOR signaling seen in the RPPA, and may in part explain the difference seen between the trametinib and cediranib treated tumors in the in vivo model. However, the lack of synergy in the in vitro experiments suggests that these findings may be driven by differences in the microenvironment rather than solely changes in the cancer cells themselves.

As the interplay between the vascular system and immune systems is now well described^34^, a potential role for immune cells in the in vivo model is also possible. Indeed, recent reports identified macrophages^35^ and B-lymphocytes^36^ as relevant in sarcoma biology. A subset of angiosarcomas are known to have high tumor mutation burden suggesting that they are immunogenic^3,37^; however, the SVR model was in nude mice and did not contain T-cells. Similar to the general lack of knowledge of the relationship between angiosarcoma tumor cells and normal endothelial cells, the spatial relationship of angiosarcoma tumor cells and immune cells is also poorly understood. Limited reports have identified high rates of infiltrating immune cells including macrophages in clinical angiosarcoma samples^29,38^. Indeed, macrophages are well known to modulate the cancer microenvironment and impact response to VEGF pathway inhibition in other tumor types^39,40^ and have been specifically associated with angiosarcoma behavior^41^. MAPK activation mediated through ERK phosphorylation is an important step in differentiation of pro-tumorigenic M2 macrophages^42^. GSEA revealed enrichment for gene sets associated with innate immunity in trametinib treated tumors compared with tumors treated in the combination, suggesting that the combination may be more effective by modulating the immune system rather than a direct effect on the cancer cells themselves. Interestingly, we also saw enrichment in several gene sets associated with T-cells in the control tumors in spite of the lymphocyte deficient model used, consistent with the recently described role of the adaptive immune system in subsets of angiosarcoma^29^. Single cell RNA-seq would potentially resolve the question of what specific changes are occurring in the microenvironment an in what cells.

One limitation of this work is the limited availability of angiosarcoma models with which to perform experiments. There is a need for syngeneic models with the same mutation patterns as their human angiosarcoma counterparts, or patient derived xenografts. No such models were available for our use. In the course of the experiments presented here, we were unable to successfully propagate the human derived cell lines in vivo in immunodeficient mice. Published in vivo models of angiosarcoma include murine knockout of FoxO^43^, TSC1^44,45^, and Notch1^46,47^. Dll4 inhibition induces vascular tumors consistent with angiosarcoma in murine models^48^. These alterations are not commonly seen in human angiosarcoma and thus are of questionable benefit for understanding the human disease.

## Conclusion

These results demonstrate that combined VEGFR and MEK inhibition is a promising treatment strategy for angiosarcoma. The difference in combinatorial effect seen in vitro versus in vivo highlights the potential importance of the microenvironment in angiosarcoma. These data support further investigation of rational targeted combinations in angiosarcoma and the development of syngeneic mouse models of angiosarcoma to better understand the potential contribution of the microenvironment in angiosarcoma pathogenesis.

## Supplementary Information

Supplementary Information.

## Abbreviations

BRAF: B-Raf proto-oncogene
DUSP: Dual specificity protein phosphatase
DMEM: Dulbecco's modified eagle medium
EGR1: Early growth response 1
ETV5: ETS variant transcription factor 5
FCGBP: Fc fragment of IgG binding protein
FGFR2: Fibroblast growth factor receptor 2
FLT4: FMS related receptor tyrosine kinase 4
GSEA: Gene set enrichment analysis
HRAS: H-Ras Proto-Oncogene
ITGA6: Integrin subunit alpha 6
IL2: Interleukin 2
IL4: Interleukin 4
IL7: Interleukin 7
KRT: Keratin
KDR: Kinase insert domain receptor
KRAS: K-Ras proto-oncogene
LGR4: Leucine-rich repeat-containing G-protein coupled receptor 4
mTOR: Mechanistic target of rapamycin
MAPK: Mitogen-activated protein kinase
MAPK1: Mitogen-activated protein kinase 1
MEK: Mitogen-activated protein kinase kinase
MYC: Myc proto-oncogene
NF1: Neurofibromin 1
NRAS: N-Ras proto-oncogene
p-ERK: Phospho-extracellular signal-regulated kinase
PI3K: Phosphoinositide-3-Kinase
PLCG1: Phospholipase C gamma 1
PCA: Principal component analysis
PCDH19: Protocadherin 19
AKT: Protein kinase B
CRAF: Raf-1 proto-oncogene
RPPA: Reverse phase protein array
SERTAD4: SERTA domain-containing protein 4
VEGFR: Vascular endothelial growth factor
