# Supplementary material for: Combined VEGFR and MAPK pathway inhibition in angiosarcoma
Source: Sci Rep. 2021 Apr 30;11:9362. doi: 10.1038/s41598-021-88703-9 (PMC8087824; doi:10.1038/s41598-021-88703-9)
Supplement: Supplementary file 1 — Supplementary Information. [file 41598_2021_88703_MOESM1_ESM.docx]

Supplemental Figures

**Combined VEGFR and MAPK pathway inhibition in angiosarcoma**

Michael J. Wagner, ^1,2^  Yasmin A. Lyons, ^3^ Jean H. Siedel, ^3^ Robert Dood ^3^ Archana S. Nagaraja,^3^ Monika Haemmerle,^3,#^ Lingegowda S. Mangala, ^3^ Pritha Chanana,^4^ Alex J. Lazar, ^5^ Wei-Lien Wang,^5^ Vinod Ravi, ^6^ Eric C. Holland, ^7^ Anil K Sood^3^

Running Title: VEGFRi and MEKi for angiosarcoma

Keywords: Angiosarcoma, MEK, MAPK, VEGFR, angiogenesis

^1^Division of Medical Oncology, University of Washington

^2^Clinical Research Division, Fred Hutchinson Cancer Research Center

^3^Department of Gynecologic Oncology & Reproductive Medicine and Center for RNA Interference and Non-Coding RNA, UT MD Anderson Cancer Center

^4^Bioinformatics Shared Resource, Fred Hutchinson Cancer Research Center

^5^Department of Pathology, UT MD Anderson Cancer Center

^6^Sarcoma Medical Oncology, UT MD Anderson Cancer Center

^7^ Division of Human Biology, Fred Hutchinson Cancer Research Center

#Current address: Martin-Luther University Halle-Wittenberg, Medical Faculty, Institute of Pathology, Section for Experimental Pathology, Halle (Saale), Germany

*Corresponding author:

Michael J Wagner

825 Eastlake Ave E

Seattle, WA 98109

[wagnermj@uw.edu](mailto:wagnermj@uw.edu)


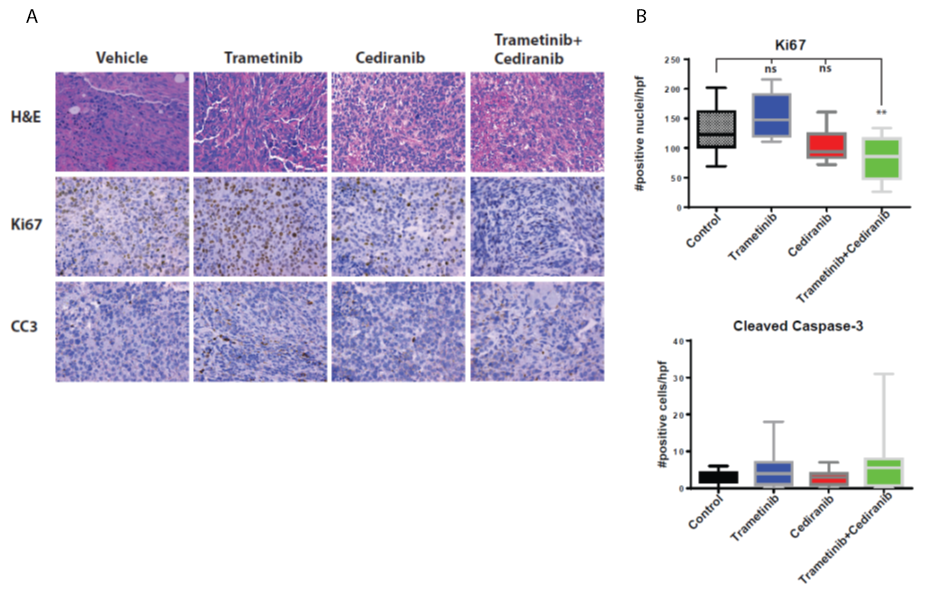


**Supplemental Figure 1.** Ki67 and Cleaved Caspase 3 (CC3) of angiosarcoma xenografts after treatment with cediranib, trametinib, and combination treatment. A. H&E and Immunohistochemistry shows significant decrease in proliferation in the combination group compared with either treatment alone as assessed by Ki67 immunohistochemistry. There were no statistically significant changes in CC3 in any of the *in vivo* treated tumors, suggesting that the observed effect is not by induction of apoptosis. B. Quantification of Ki67 and CC3 staining.

Graphs were made in GraphPad Prism 7 (<https://www.graphpad.com/scientific-software/prism/>). Unedited/cropped images were formatted in Adobe Photoshop 2020 (https://www.adobe.com/) to create the figure layout.

**Supplemental Figure 2. Reverse phase protein array (RPPA) results of apoptosis and mitotic markers in HAMON cells after 72 hours of treatment. A.** BIM expression was increased compared with control in the single agent treatment groups (trametinib p = 0.006, cediranib p = 0.004) and in the combination group (p = 0.001). Beclin expression was increased compared with control in the single agent treatment groups (trametinib p <.001, cediranib p = 0.04) and in the combination group (p < 0.001). Cleaved caspase-7 was increased compared with control in the combination group only (p < 0.001). **B.** Cyclin D3 expression increased with combination treatment only (p < 0.001). Cyclin B1 decreased in all treatment groups (trametinib p =0.003, cediranib p = 0.01, combination p = 0.002). 4E-BP1 S65 phosphorylation increased in all treatment groups (trametinib p = 0.03, cediranib p = 0.04, combination p = 0.004). *p ≤0.05, **p≤0.01, ***p≤0.001.


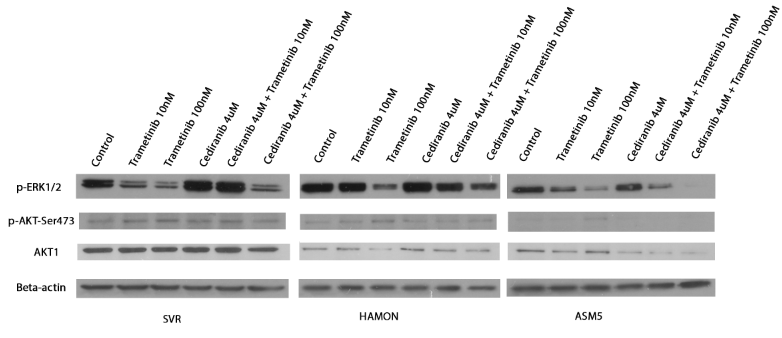


**Supplemental Figure 3.** Western blot of angiosarcoma cell lines for p-ERK, p-AKT, and total AKT. Total ERK for HAMON and ASM5 cell lines under the same treatment conditions is seen in Supplemental Figure 4.

Supplemental Figure 3 Original Western Blot Images:

**Supplemental Figure 4. Total ERK expression of ASM5 and HAMON cell lines.**
